# Spatial Accessibility and Equity Evaluation of Medical Facilities Based on Improved 2SFCA: A Case Study in Xi’an, China

**DOI:** 10.3390/ijerph20032076

**Published:** 2023-01-23

**Authors:** Linggui Liu, Yi Zhao, Han Lyu, Sining Chen, Yuheng Tu, Shuyun Huang

**Affiliations:** School of Humanities and Social Science, Xi’an Jiaotong University, Xi’an 710049, China

**Keywords:** medical facilities, spatial accessibility, two-step floating catchment area (2FSCA), ArcGIS

## Abstract

Accurate evaluation of the accessibility of medical facilities is a prerequisite for the reasonable allocation of medical resources in a city. The accessibility of medical facilities depends not only on the distance to the supply and demand points, but also on the time spent in the process, and the supply capacity of the supply points. Taking Xi’an City of Shaanxi Province as an example, this paper comprehensively considers the facility supply capacity and introduces the selection probability function based on the two-step floating catchment area (2SFCA) method. In addition, in order to approximate the residents’ acceptance of different types of hospitals for long-distance medical treatment in real situations, different levels of search radius were set for the different levels of hospitals, and ArcGIS was used to measure the accessibility and evaluate the spatial layout of medical facilities in the main urban area of Xi’an. The results show that there is a significant difference in the accessibility of medical facilities in the main urban area of Xi’an, and the accessibility tends to decrease gradually from the central city to the periphery. The inequity in the allocation of medical facilities in the main urban area of Xi’an is more obvious, with about 81.64% of people having access to 54.88% of medical resources. The accessibility evaluation model established by the improved 2SFCA method can obtain more accurate and objective evaluation results. This study can provide a reference basis for urban medical facilities’ planning and rational spatial layout.

## 1. Introduction

In recent years, China’s urban space has entered the stage of high-quality development, gradually changing from focusing on expanding urban physical space to improving residents’ quality of life, so the scientific configuration of public service facilities is of important research value. Medical services, as one of the basic public services, are directly related to people’s quality of life, while the Corona Virus Disease 2019 (COVID-19) pandemic that started in recent years has also made people pay more attention to the quality of public medical services. Under these circumstances, improving medical services and allocating urban public medical resources more scientifically and effectively has gradually become a research problem of wide concern.

Compared with other countries, China has a large population base and a higher demand for medical resources. At present, for the whole country, there is still an uneven spatial allocation of medical resources and unbalanced development [1]. The insufficient number of high-grade medical facilities and the low level of medical facility configuration in many cities has led to the phenomenon of difficult and crowded access to medical care, which requires increased investment in medical resources and expansion of high-quality medical resources. In turn, the radiation range of medical resources should be expanded to solve the phenomenon of unbalanced spatial allocation of medical resources and promote their rational distribution. Accessibility-related research is essential for optimizing the layout of public service facilities and achieving service equality. Therefore, in the study of medical resource allocation, accessibility evaluation has also received great attention from scholars.

Accessibility refers to the convenience of getting from one point to another [2], and is an important indicator to assess whether the allocation of public service resources is reasonable. In medical and health services, accessibility has also gradually developed into a research hotspot in the field of medical geography, indicating the degree of superiority or inferiority between the supply and demand points of medical services [3]. It can reflect the inefficient spatial allocation of scarce medical facilities and the inequitable spatial distribution of medical resources in traditional planning [4]. Therefore, it is important to measure the spatial accessibility between medical service facilities and residential locations by studying the actual situation of the two for rational allocation of medical resources, improving people’s quality of life, and maintaining social stability. Due to the large population base and uneven distribution of medical resources in China, it is necessary to update the accessibility research calculation models in a timely fashion in order to make the research results more consistent with the actual environment and thus improve the efficiency and equity of medical resource utilization.

Hansen first introduced the concept of accessibility in 1959 and studied the relationship between urban land used and accessibility using the gravity method [5]. The study of accessibility has thus gained the attention of many scholars, who have widely applied the accessibility theory to the spatial layout of public service facilities and the study of fairness. Spatial accessibility of public service facilities can be broadly understood as the ease of access to facilities and services by means of transportation and roads and is mainly concerned with the “quality” and “quantity” of public services affecting a certain area [6]. In the accessibility studies of medical facilities, the number of beds and medical personnel is often used to reflect the accessibility of medical facilities. Different research methods are used in accessibility studies according to different research subjects [7]. In recent years, several modeling methods have been applied to measure the spatial accessibility of urban healthcare resources, among which the two-step floating catchment area (2SFCA) method, potential model, and gravity model are common. Since the potential model ignores the difference in travel impedance of residents to different levels of hospitals, it is not consistent with reality [8]. The gravity model mainly considers the competition effect among medical facility points, and the reliability of this measure is somewhat questioned [9]. In addition, since the two-step floating catchment area method incorporates the interaction between supply, demand, and minimum cost into the measure of accessibility, it has advantages in the equity measure of medical resources [10]. The two-step floating catchment area method approach is the most widely used in the evaluation of spatial accessibility of public service facilities.

The traditional two-step floating catchment area method has two drawbacks: the use of a dichotomous method to deal with distance decay and the use of a single search radius. In order to solve the defects of the dichotomous method of the two-step floating catchment area method to deal with distance, many scholars introduced the distance decay function in the original model, forming an improved two-step floating catchment area method mainly in the form of exponential function [11], Gaussian function [12], kernel density function [13] and so on. For the expansion of the search radius, the main forms are variable radius [14], dynamic radius [2], multilevel radius [15], and nearest facility type [16]. In addition to this, there are two other improvements to the two-step moving search method model, the first of which is the extension of demand or supply competition. Wan et al. (2013), after pointing out the competition effect between facilities when multiple facilities exist within the search radius of a demand point, proposed 3SFCA by adding a third step to the search including all demand points and facilities, in which the selection weight between demand point i and facility j is calculated [17]. Luo (2014) introduced the classical Huff model (Huff model) to portray the selection weight variable, thus proposing Huff 2SFCA as an extended form [18]. Based on the classification of hospitals, Tian et al. (2022) proposed an attraction model determined by the number of doctors and annual outpatient visits, and proposed a tolerance decay function to replace the distance decay function based on this model [19].The second aspect is the extension of travel mode, and Fransen et al. (2015) proposed a Commuter-based 2SFCA to consider the impact of commuting behavior on public service accessibility [20]. Tao and Wang (2022) aimed to measure transportation-induced healthcare inequity in Shenzhen by comparing the accessibility of medical facilities under different transportation situations [21]. Xing and Ng (2022) adopted the modal accessibility gap (MAG) index to reveal the spatial disparities of accessibility between public transport and private car modes. In addition, a new travel cost calculation method is proposed, which takes monetary cost into account for the first time [22].

Nowadays, although accessibility-related studies are still improving, there is a lack of consideration of the hierarchical characteristics of medical facilities in existing domestic studies. There are differences in the service scope of different levels of medical facilities, and the results of accessibility studies that consider all medical facilities as having the same characteristics may be subject to large errors [1]. In addition, traditional spatial accessibility studies are only spatial, and their fundamental purpose is to assess the ease of reaching a destination, which does not meet the needs of an increasingly developed transportation system, where in practice traffic conditions can vary significantly in time and space [23]. Many existing accessibility studies use traditional static road network data, and some of them estimate the actual travel time by the grade and time period average speed of road sections, but they do not consider the impact of actual traffic conditions on the time cost. Moreover, it has been suggested that not all patients need to be hospitalized when they are ill, and the number of hospital patients and beds should be considered together when calculating hospital service levels [24]. In addition, the supply of medical facilities in previous studies is often summarized using the number of hospital beds or the number of health technicians, and the estimation of the supply of medical facilities is too homogeneous. In view of the above problems, the specific improvements of the medical facility accessibility model in this study are as follows: (1) different travel thresholds are set for different levels of medical facilities. In addition, the distance and time of the search radius are crawled through Python to obtain the shortest driving travel time and its corresponding driving distance in Gaode Map. (2) The supply capacity of medical facilities is considered comprehensively, and factors such as the proportion of patients hospitalized, the number of health technicians, and the number of beds are included in the calculation of supply capacity. (3) A selection probability function is introduced into the model to take into account the influence brought by the quality of facility services on the residents’ choice of medical treatment. At the spatial level, the accessibility evaluation model of urban medical facilities is constructed with the help of ArcGIS 10.4 software to evaluate and analyze the accessibility of the current spatial layout of medical facilities in Xi’an; at the data level, the parity evaluation of medical facilities in Xi’an is carried out based on the Lorentz curve and Gini coefficient. This study analyzes and evaluates the accessibility results through different quantitative methods, hoping to provide methodological references for related academic studies and decision-making references for the government to seek a reasonable spatial layout of medical services.

## 2. Methods and Data Source

### 2.1. Accessibility Measurements: Two-Step Floating Catchment Area (2FSCA)

2SFCA was first proposed by Radke and Mu (2000) to represent the spatial accessibility of demand points by calculating the supply demand ratio based on supply and demand points [25]. Luo and Wang (2003) applied the two-step floating catchment area method to the spatial accessibility study of primary health care in the 10-county Chicago area to assess the variation of spatial accessibility of primary health care in the Chicago area and achieved better results [26]. Hu et al. (2012) concluded that the two-step floating catchment area method can effectively show the spatial variation of health care accessibility, so the two-step floating catchment area method was used to measure the spatial accessibility of health care in villages in Donghai County, Jiangsu Province, and to comprehensively study the distribution characteristics of areas lacking medical care [4].

Since the two-step floating catchment area method takes into account the threshold constraints and introduces the supply demand ratio index, it can well reflect the actual situation of urban residents’ choice of nearby facilities and effectively show the spatial variation of medical accessibility and is currently the main research method for measuring spatial accessibility in the field of health care, and its basic idea is divided into two steps.

In the first step, for each supply point *j*, search all demand points (*k*) whose distance *j* is within the search radius (*d*_0_) and calculate the supply demand ratio *R_j_*.

(1) $$R_{j}=\frac{S_{j}}{\sum_{k\in \left[d_{kj}\leq d_{0}\right]\;}P_{k}}$$

where *S_j_* denotes the total supply of supply point *j*, mostly measured by indicators such as the number of beds or the number of health technicians, *d_kj_* denotes the distance from point *k* to point *j*, *P_k_* is the demand of demand point *k* within the search radius, mostly measured by the number of population, and *R_j_* is the supply-to-demand ratio of point j, which is the service capacity of that supply point.

In the second step, for each demand point *i*, search all the supply points (*j*) whose distance *i* is within the search radius (*d*_0_), and sum up all the supply and demand ratios *R_j_* to get *A_i_^F^*, the accessibility of point *i*.

(2) $$A_{i}^{F}=\underset{j\in \left[d_{ij}\leq d_{0}\right]}{\sum }R_{j}=\underset{j\in \left[d_{ij}\leq d_{0}\right]}{\sum }\frac{S_{j}}{\sum_{k\in \left[d_{kj}\leq d_{0}\right]\;}P_{k}}$$

where *A_i_^F^* indicates the accessibility of demand point *i*, *R_j_* is the supply demand ratio, or service capacity, calculated for hospital *j* within the travel threshold for medical care, *d_ij_* is the distance between demand point *i* and supply point *j*, and *P_k_* is the total number of people at point *k* within the search range. The larger the calculated *A_i_^F^*, the better the accessibility of location *i*.

### 2.2. Optimize the Two-Step Floating Catchment Area

The traditional two-step floating catchment area method ignores the fact that the variation of spatial distance within the search domain affects residents’ choice of facilities, which is inconsistent with reality. Based on this, scholars have introduced distance decay functions to extend the traditional two-step floating catchment area method to address the shortcomings of consistent accessibility in the search domain. The commonly used distance decay functions are the power function, exponential function, Gaussian function, etc. In practical applications, the form of the distance decay function should be selected according to the characteristics of the facility’s usage behavior. People’s medical behavior can be approximated by mathematical functions to a Gaussian function that decays gradually with increasing distance or time, and the decay rate is accelerated by slow, then slowed down. In addition, in recent years, the two-step floating catchment area method using the Gaussian function as a distance decay function has been widely used in medical facility accessibility research at home and abroad.

In this paper, the distance decay function in the form of the Gaussian function is used:

(3) $$\begin{array}{c} f\left(d_{ij},d_{0}\right)=\frac{e^{-\left(1/2\right)\times {\left(d_{ij}/d_{0}\right)}^{2}}-e^{-\left(1/2\right)}}{1-e^{-\left(1/2\right)}} \end{array}$$

After adding the Gaussian function, the two-step floating catchment area model expression is:

(4) $$R_{j}=\frac{S_{j}}{\sum_{k\in \left[d_{kj}\leq d_{0}\right]\;}P_{k}f\left(d_{kj}\right)}$$

Unlike other public facilities, different levels of medical facilities have different service capacities. Some scholars point out that the service radius of a hospital is an important indicator of the quality of medical care, service capacity, and the size of the scope of the hospital [27]. In practice, people believe that the higher the grade of the hospital, the higher the relative technical level of the doctors, so people choose to seek medical treatment with a greater range of acceptance of the distance to the hospital with a higher grade. In order to reflect the influence of different grades and sizes of medical facilities on residents’ access to medical care, this paper sets different search radii for different grades of medical facilities. The search radius for reaching tertiary, secondary, and sub-secondary hospitals by driving is set to 60, 45, and 30 min, respectively.

For *S_j_*, the medical facility supply capacity in the formula, the number of beds is an important indicator of the development level of health resources in a country or region, and most studies have calculated the number of beds in medical facilities as the medical facility supply capacity [28]. Some scholars believe that the number of health technicians can better represent the supply capacity of medical facilities, especially when hospitals do not have beds [29]. Some scholars also believe that the number of hospital patients and the proportion of hospitalization should be considered comprehensively when calculating the supply level of hospitals, and the number of health technicians and the number of beds in hospitals should be considered comprehensively. In addition, the improved hospital supply capacity model was proposed according to the Hospital Grading Management Standard [30]. In a comprehensive comparison, the improved hospital supply model is more representative of the hospital supply capacity, so the improved supply capacity model is used in this paper as follows:

(5) $$S_{j}=B_{j}\times U_{j}\times W_{j}\times L_{j}$$

where *S_j_* is the supply level of the medical facility, indicating the overall service level of the medical facility. *B_j_* is the number of beds in the medical facility, *U_j_* is the utilization rate of beds in the medical facility, *W_j_* is the ratio of health technicians per bed, and *L_j_* is the number of inpatients per 100 visits.

In addition to this, when multiple facilities exist within the search radius of a demand point, there is a competition effect between facilities [17]. The traditional two-step floating catchment area method assumes that the demand for all facility points does not vary with the number of facility points, leading to an overestimation of the demand for certain facility points [31]. Real-life hospitals’ service quality, location, and supply can all have an effect on competition among hospitals. Therefore, competition among medical facilities was considered in the improved model, and the Huff model was used to calculate the probability of selecting a medical facility for the population in each residential location.

The expression is:

(6) $$Prob_{ij}=\frac{S_{j}f\left(d_{ij}\right)}{\sum_{k\in \left[d_{ik}\leq d_{0}\right]}S_{k}f\left(d_{ik}\right)}$$

*Prob_ij_* is the probability of *i* choosing *j*; *S_j_* is the level of medical facility supply, and *f*(*d_ik_*) is the decay function of the distance between *i* and *k*.

The improved two-step floating catchment area method is calculated as follows:

Step 1, for each medical facility:

(7) $$R_{j}=\frac{S_{j}}{\sum_{k\in \left[d_{kj}\leq d_{0}\right]}P_{k}f\left(d_{kj}\right)Prob_{kj}}$$

Step 2, for each settlement:

(8) $$A_{i}^{F}=\underset{j\in \left[d_{ij}\leq d_{0}\right]}{\sum }Prob_{ij}f\left(d_{ij}\right)\frac{S_{j}f\left(d_{ij}\right)}{\sum_{k\in \left[d_{kj}\leq d_{0}\right]}P_{k}f\left(d_{kj}\right)Prob_{kj}}$$

### 2.3. Overview of the Study Area

**Figure 1:**
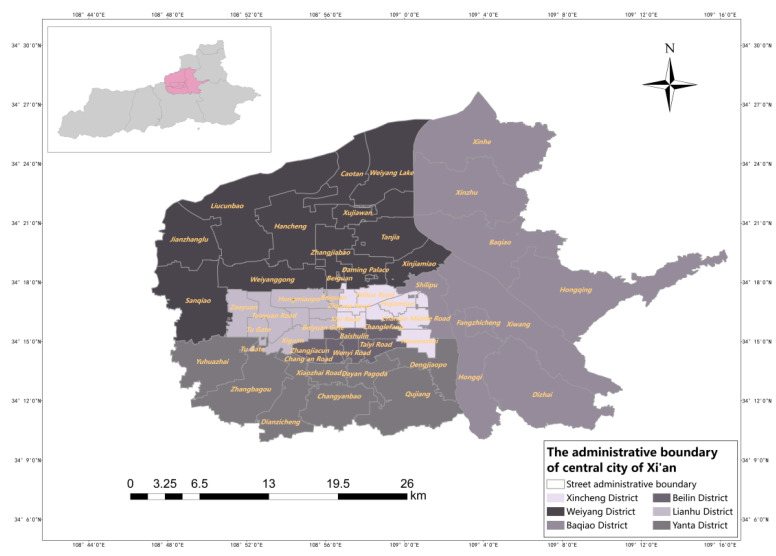
The administrative boundary of central city of Xi’an.

Xi’an is the capital of Shaanxi Province and is an important central city in western China, and as of the end of 2021, Xi’an Xi’an City has 11 districts and 2 counties under its jurisdiction. In this study, the main urban area of Xi’an is selected as the study area, with a total area of about 843.56 km^2^, accounting for 16.17% of the city’s area, and 53 streets and towns, including a total of six administrative districts, namely, Beilin District, Weiyang District, Xincheng District, Yanta District, Baqiao District and Lianhu District; an overview of the study area is shown in Figure 1. According to the data of the seventh population census published by the Xi’an Bureau of Statistics, the resident population of Xi’an is 12,952,500, an increase of about 4.48 million compared with the sixth national census, with an average annual increase of 4.34%, of which the resident population in the main urban area is about 4.95 million, accounting for 38.22% of the resident population in Xi’an. This shows that the resident population of Xi’an is increasing year by year, and the main urban area is densely populated and has a high demand for medical resources. The tertiary hospitals such as Xi’an Central Hospital and Xi’an Ninth Hospital in the main city are often overcrowded, and many patients go to tertiary hospitals if they have to seek medical treatment, even if they are far away. The blindness of patients in seeking medical treatment reflects the unreasonable allocation of medical resources, and it is especially critical to accurately evaluate the allocation of medical resources [32].

### 2.4. Data acquisition and Preprocessing

#### 2.4.1. Medical Facility Data

Since different levels of medical facilities have different attractiveness to residents for medical treatment, the higher the level of the hospital, the more likely it will attract residents from a greater distance. In this paper, hospitals of different grades in the main urban area of Xi’an are selected as the research object.

**Figure 2:**
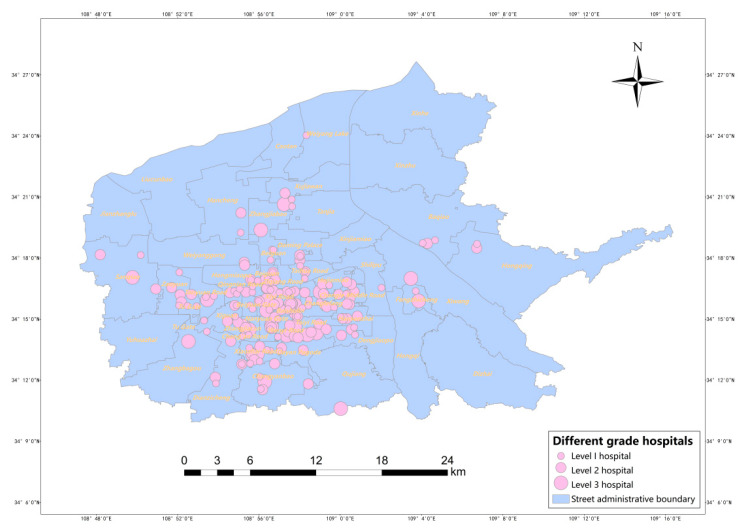
Distribution of different-grade medical facilities.

This paper crawled the information on the names, addresses, types, grades, and the number of beds of medical facilities in the main urban area of Xi’an from a database of the 91 hospitals. Since the data obtained from the 91-hospital database lacked spatial information, this paper converted the detailed structured addresses into WGS-84 latitude and longitude coordinates through the geocoding interface of Gaode Map and coordinate conversion tools to supplement the spatial information data of medical institutions. Then, statistics on the number of consultations, admissions, and bed utilization rates of different levels of medical institutions were obtained from the 2019 Xi’an Health and Health Development Statistical Bulletin, which was used to comprehensively estimate the service capacity of different levels of hospitals. The final data were obtained for 187 hospitals, including 27 tertiary hospitals, 84 secondary hospitals, and 76 primary and sub-primary hospitals; the distribution of medical facilities is shown in Figure 2.

**Figure 3:**
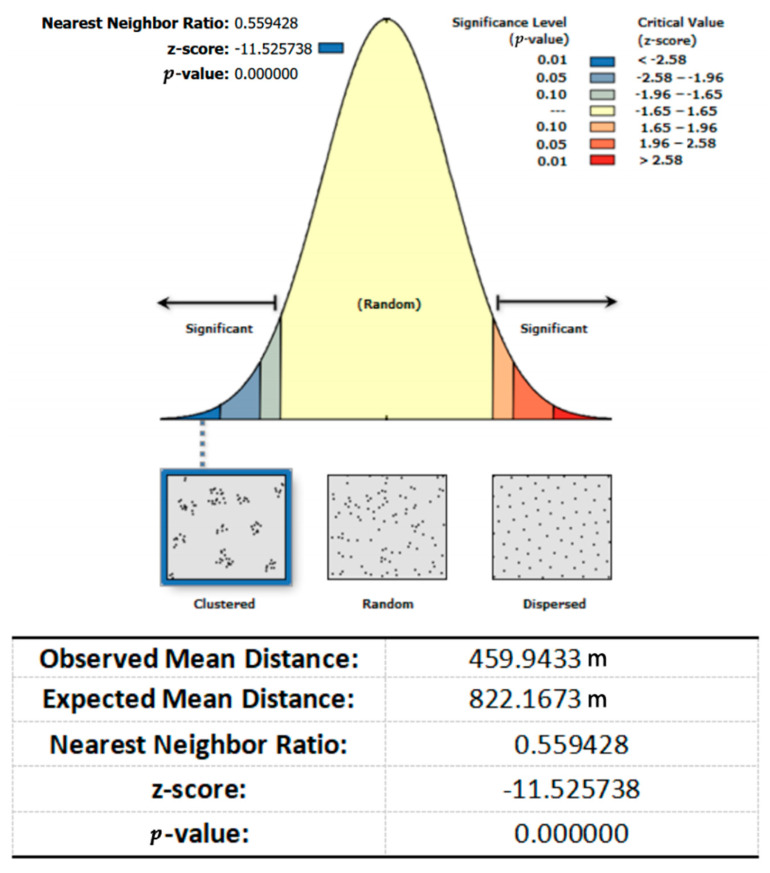
Nearest neighbor result reports.

This paper analyzes the spatial clustering of medical facilities in the main urban area of Xi’an by using the “mean nearest neighbor” tool in ArcGIS. The results of the spatial clustering of medical facilities in the main urban area of Xi’an are shown in Figure 3. The results show that the nearest neighbor index is less than 1, the z-value is less than −2.58, and the *p*-value is less than 0.01, indicating that the medical facilities in the main urban area of Xi’an are clustered and distributed.

#### 2.4.2. Population Data

Most of the existing studies on the accessibility of medical facilities have been conducted at the street scale, taking the center of mass of the street as the demand point of medical facilities, and the accessibility values obtained in this way are not accurate enough. In this paper, we try to take the spatial location of the residential district as the demand point of medical facilities from the population of the district. Due to the lack of population data at a smaller scale than the street, the number of households in the neighborhood in the same street is summed up, and the ratio of the total population of the street multiplied by the number of households in the neighborhood to the total number of households in the street is taken as the population of the neighborhood. The number of households in the neighborhood and the latitude and longitude coordinates were obtained by python crawling to a total of 856 residential neighborhoods, and the distribution data of population in each neighborhood were obtained from the Seventh National Census Report of Xi’an City.

**Figure 4:**
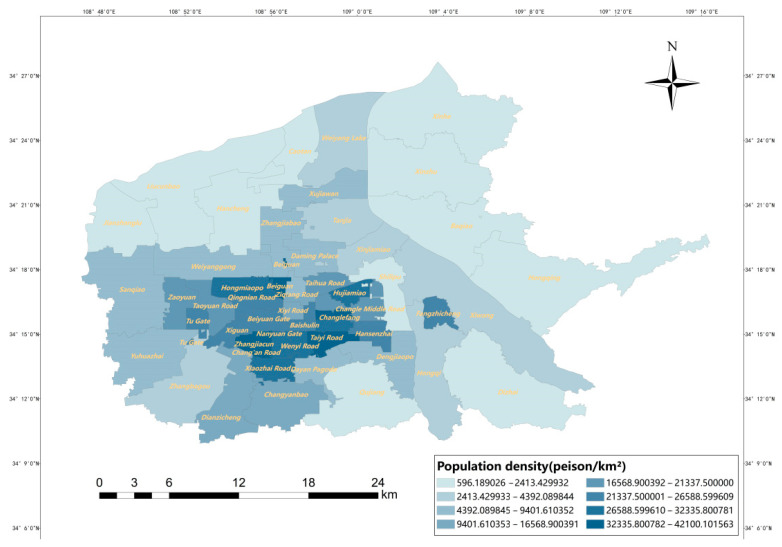
Population density.

**Figure 5:**
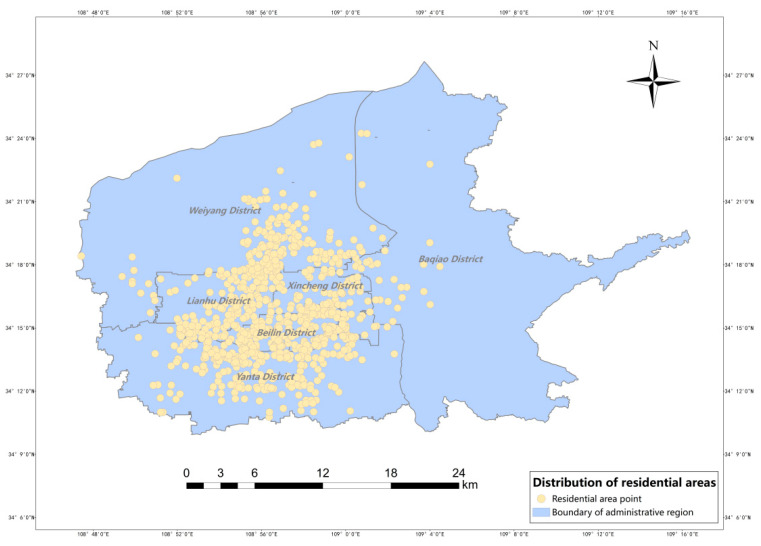
Distribution of residential areas.

The distribution of population density in the main urban area of Xi’an and the distribution of residential neighborhoods in the main urban area of Xi’an were obtained using ArcGIS, as shown in Figure 4 and Figure 5. The results show that the population density varies widely, with the highest street reaching 42,100 people/km^2^ and the lowest northern peripheral street only 596 people/km^2^. In terms of spatial distribution, the population distribution of Xi’an shows a circular structure with central concentration, diffusion at the inner edges, and increase at the outer edges. Residential areas are mainly concentrated in the core areas of the city, with an overall core–edge distribution pattern, with Yanta, Xincheng, Lianhu, and Beilin districts having the most distribution and Baqiao district having the least.

#### 2.4.3. Obtaining Actual Transportation Costs

Driving is one of the main ways for residents to travel, with better comfort and convenience. Considering the physical condition of patients and the urgency of medical treatment, residents will give priority to driving with better comfort and convenience when going out for medical treatment. In addition, according to the data of 2021, the per capita car ownership in Xi’an ranks sixth in China, with an average of 2.73 people owning a car. Private car travel and taxi (online taxi) travel also account for the largest proportion in Xi’an. Therefore, in this paper, the driving mode is selected as the transportation mode for residents to go for medical treatment, by taking the actual driving time between supply and demand points as the transportation cost to calculate the accessibility. The specific operation was to crawl the data of driving time and distance from the Gaode Map API website through Python.

**Figure 6:**
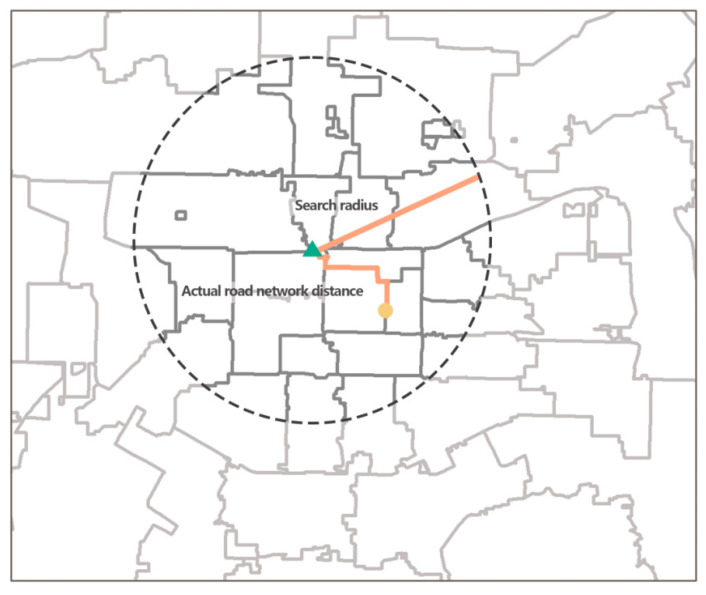
Buffer radius.

In order to reduce unnecessary calculations, the OD cost matrix is first built through the Network Analyst module to find all the straight-line distances from 856 residential points to 187 medical facilities, followed by excluding the supply and demand points outside the reachability threshold range by building a larger buffer, as shown in Figure 6. Since the study in this paper is based on the real distance of the actual road network, the network distance between two points in this case must be greater than the straight-line distance, so the buffer radius is set as the real network distance. In this paper, the average speed of motor vehicles is set to 30 km/h in the calculation process, which is used to determine the buffer radius when constructing the OD cost matrix.

### 2.5. Technical Process

**Figure 7:**
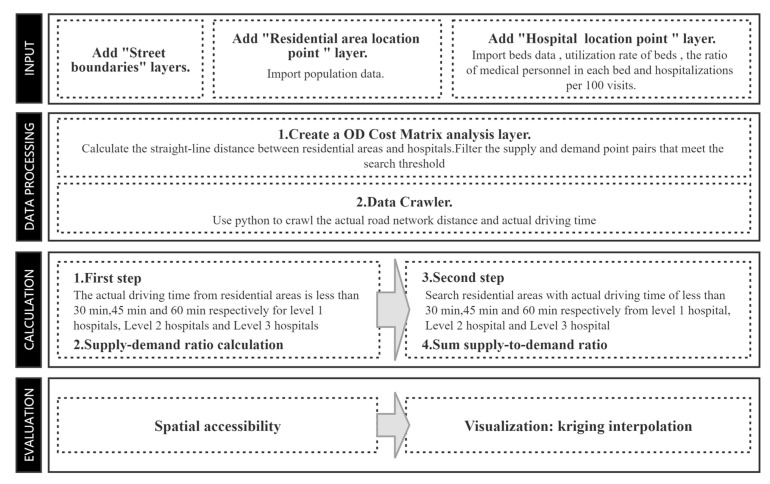
Implementation of the 2SFCA model in the Geographic Information System (GIS).

The implementation of the 2SFCA model in GIS is shown in Figure 7. The specific process is: (1) Import the spatial location information of the residents and medical points as well as the street boundaries and other information, and based on the above information, build the OD cost matrix in ArcGIS to calculate the straight-line distance from each resident point to all medical points. (2) Based on the established OD cost matrix, the actual distance and the shortest driving time of the road network are crawled in API using Python. (3) Set different search radius according to the scale of medical points, 30 min for primary medical points, 45 min for secondary medical points, and 60 min for tertiary medical points, and take medical points as the starting point to find all the residential points within the search threshold and calculate the supply demand ratio of each medical point. The sum of the supply and demand ratios of all medical points within the search threshold. (4) Assign the calculated reachability results to each administrative street, display them in ArcGIS in a hierarchical manner, and visualize them by kriging interpolation.

## 3. Results

### 3.1. Case Study

#### 3.1.1. Analysis of Accessibility from a Spatial Perspective

**Figure 8:**
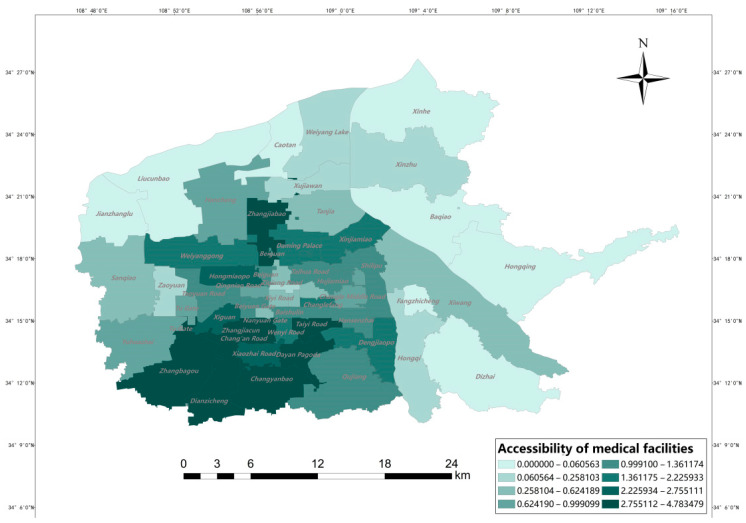
Accessibility of medical facilities.

Figure 8 shows the results of accessibility of medical facilities in each subdistrict of six urban districts in Xi ‘an, from which it can be seen that when the real network distance is used as the buffer radius, the accessibility distribution of medical facilities in the main urban areas of Xi’an is characterized by differences: the lowest value is 0.000 and the highest value is 4.783. It can be seen that the spatial accessibility distribution is not uniform. Among them, the high accessibility values are mainly concentrated in Yuhuazhai Street, Zhanbagou Street, Electronic City Street and Changyanbao Street. Although the density of medical facilities in Yanta District is not as high as that in the central city of Beilin District, Xincheng District and Lianhu District, the population density in Beilin District is high, so Yanta District has more resources per capita within the service radius of medical facilities at the street office level, which leads to the high accessibility values in Yanta District. In general, the accessibility values of medical facilities in the northwest and northeast of the city are generally low, while the central city and the south of the city are areas with high accessibility values. The central city has a high accessibility value due to its developed economy, convenient transportation, and perfect medical facilities. In addition, the accessibility of medical facilities in Xiwang Street in Baqiao District has higher values compared to other surrounding areas because the clustering of industries leads to the gathering of population, which leads to the layout of related medical facilities in this area, indicating that the convenient transportation network can help residents overcome the effects of distance decay, resulting in a high per capita occupancy of medical resources. In the northwestern area of Weiyang District, although the density of facilities is not low and the population density is not high, the accessibility values are affected by distance attenuation, and thus the accessibility values are low. In terms of the overall effect of spatial distribution, the spatial accessibility of medical facilities in Xi’an shows a decreasing feature from the central city and the southern area to the periphery.

To accurately describe the accessibility of medical facilities within each subdistrict of the main urban area of Xi’an, the accessibility of medical facilities in residential areas can be depicted by the interpolation tool in GIS. Liu Jinsong et al. suggested that different interpolation methods should be compared based on the mathematical characteristics of the data to select the optimal one [33]. According to the comparison of four common interpolation methods in Arcgis by Yan Takeda et al. [34], when the mean error and root mean square error are the smallest overall, the interpolation effect is better, especially when the root mean square error is smaller. From Table 1, we can see that the ordinary Kriging model is the most suitable. In addition, the Kriging interpolation method takes into account the spatially correlated nature of the described objects in the process of data gridding, which makes the interpolation results more scientific and closer to the actual situation, and the Kriging variance shows that this interpolation method is more reliable.

**Figure 9:**
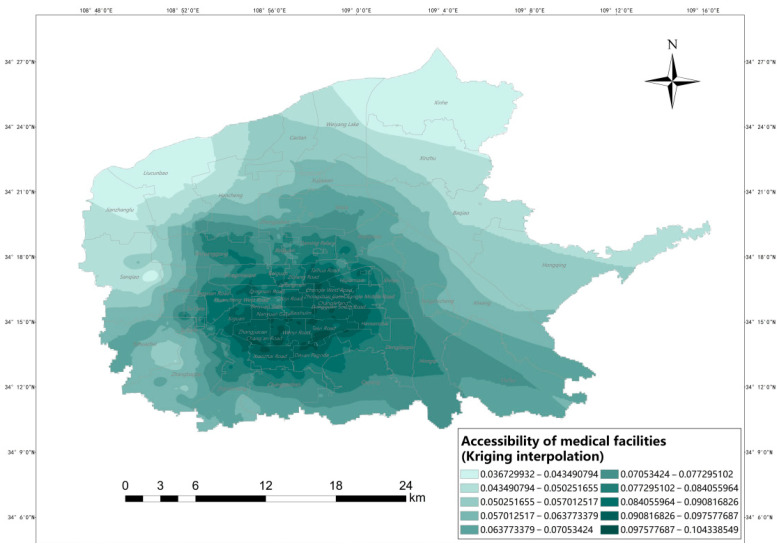
Accessibility of medical facilities (Kriging interpolation).

Figure 9 shows the results of interpolation analysis of the accessibility values of medical facilities in residential areas of the main urban area of Xi’an, the overall accessibility of medical facilities to residential areas varies significantly, with accessibility decreasing from the center to the periphery. The areas closer to the central area also have considerable accessibility. Although the central area has a large population, it also has the largest number of medical facilities. In addition, the central area is small, and the transportation time is short, so the corresponding accessibility value is high. It is worth noting that the accessibility of medical facilities in the southern part of Zhanbagou Street, Electronic City Street, and Changyanbao Street is significantly lower than that of each street in Figure 8. This is mainly due to the uneven distribution of population density, coupled with the limited scale and number of its medical facilities, which reduces the accessibility value of the southern neighborhood.

#### 3.1.2. Analyzing Accessibility from a Data Perspective

Currently, the Lorenz curve and Gini coefficient are widely used in the evaluation of the parity of public service facilities. There are many kinds of Gini coefficient calculation formulas, and the more frequently used and classical formulas are as follows:

(9) $$Gini=1-\overset{n}{\underset{i=1}{\sum }}\left(X_{i}-X_{i-1}\right)\left(Y_{i}-Y_{i-1}\right)$$

where *Gini* is the Gini coefficient, where *X_i_* (*i* = 1, 2...n) is the cumulative percentage of the population in the corresponding unit, and *Y_i_* (*i* = 1, 2...n) is the cumulative ratio of resources in the corresponding unit. The correspondence between the values of the Gini coefficient and equity are shown in Table 2.

**Figure 10:**
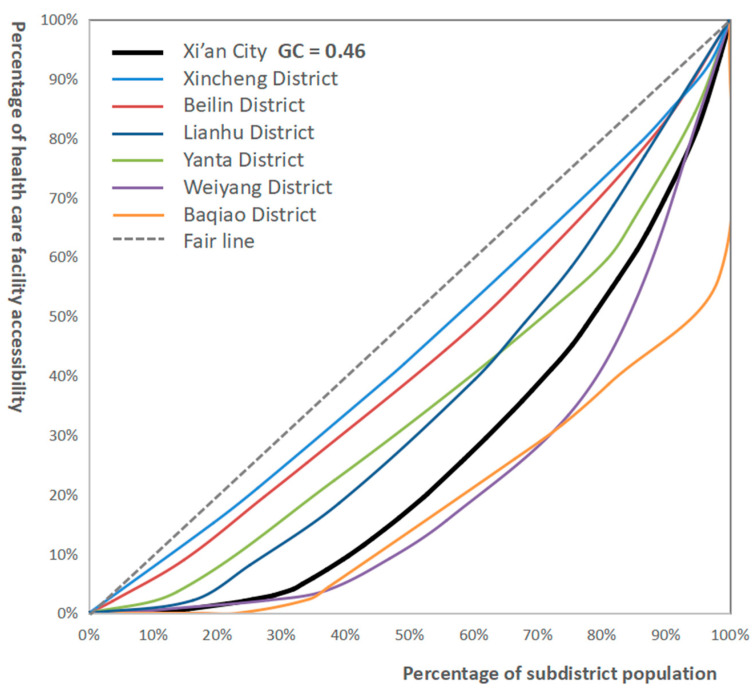
Lorenz curve and Gini coefficient of medical facilities.

Based on the accessibility of medical facilities and the distribution of street population, in this paper, the Lorenz curves of the central urban area of Xi’an and six administrative districts are drawn, and the corresponding Gini coefficients are calculated, as shown in Figure 10 and Table 3. In general, the Gini coefficient of the accessibility of medical facilities in the central urban area of Xi’an is 0.46, indicating obvious spatial inequality. About 81.64% of people in the central urban area of Xi’an can enjoy 54.88% of medical resources. The Gini coefficient of Beilin district and Xincheng district is less than 0.2. The Lorentz curve is closest to the fair line, and the distribution of internal resources is the most fair. The second is the southern periphery of Lianhu district and Yanta district, its internal medical resources distribution is relatively fair; However, Weiyang district and Baqiao district located in the northern periphery have significant differences in the allocation of medical facilities, especially in Baqiao district, where nearly 25% of the population cannot access medical resources.

### 3.2. Model Comparison

**Figure 11:**
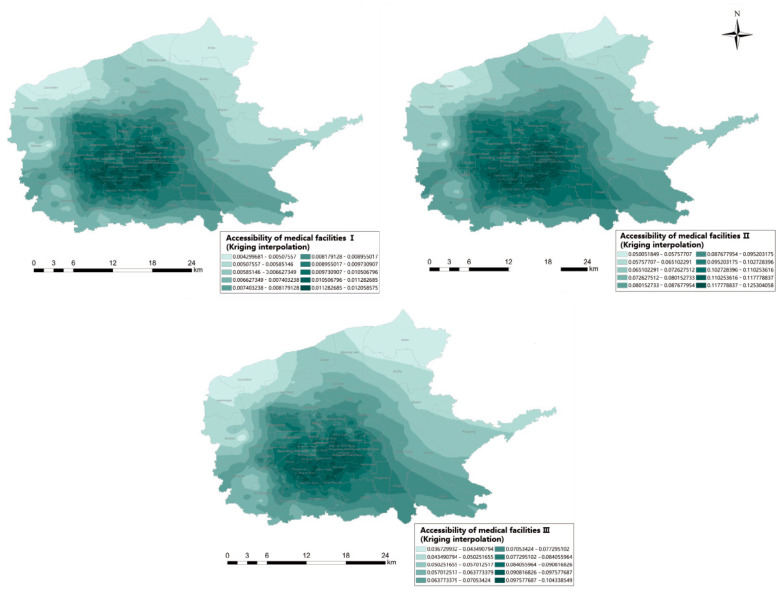
Comparison of accessibility.

As shown in Figure 11 and Table 4, the mean of regional accessibility was 0.010 without considering the comprehensive supply capacity of hospitals and the principle of preferential selection, but 0.107 and 0.08 after considering the comprehensive supply capacity of hospitals and the principle of preferential selection, respectively. The accessibility range of the former was 0~0.126, and the latter was 0~0.104. The accessibility range was narrowed, and the standard deviation was slightly reduced. However, the standard deviation increased compared with that before the optimization, indicating that the accessibility difference between regions is more obvious when considering the comprehensive supply level of medical institutions instead of simply representing the supply capacity of medical institutions by beds. This reflects the fact that the uneven distribution of medical institution resources in the region appears more serious than it actually is.

## 4. Discussion

### 4.1. Advantages of the Improved Model

This paper improves the accessibility evaluation model of medical facilities in Xi’an, and quantitatively evaluates and graphically expresses the accessibility of medical facilities with the help of spatial analysis and visualization functions of GIS. The approach adopted in this paper has the following advantages: (1) For medical facilities, it is crucial for residents to have timely and efficient access to medical resources, so the introduction of the probability function makes the model closer to reality and the results more accurate, which facilitates the spatial layout planning of medical facilities at the macro level, considering that residents give priority to service quality and proximity to their homes in real situations. (2) The search radius involves distance decay, which provides more accurate calculation results by combining the real road network and the actual minimum time and distance along the route derived from API path planning. (3) Unlike other public service facilities, the scientific evaluation of the comprehensive supply capacity of medical facilities is particularly important in the actual evaluation. Therefore, we adopt an improved supply model, which is more relevant to the problem of evaluating the accessibility of medical facilities, taking into account the competitiveness among facilities, as well as important factors such as the number of beds in medical facilities, the utilization rate of beds, and the ratio of health and technical personnel per bed. (4) The accessibility results are analyzed from both spatial and data perspectives. At the spatial level, with the help of ArcGIS, the accessibility evaluation results are expressed in the form of maps and kriging interpolation by street offices, which can comprehensively show the accessibility spatial distribution characteristics of medical facilities; at the data level, the Lorentz curve and Gini coefficient are combined, and can more intuitively reflect the fairness of medical facilities distribution. 

### 4.2. Shortcomings of the Improved Model

The results of this paper can help to understand the spatial accessibility of medical facilities in the central city of Xi’an and provide a basis for the relevant departments to make reasonable planning and layout. For example: (1) The setting of the maximum travel time threshold for residents of different levels of medical facilities is somewhat subjective, and the next study can conduct sensitivity analysis on accessibility by setting different time impediments. (2) The paper does not consider the diversity of travel modes of residents when seeking medical treatment, and uniformly adopts the transportation mode of driving. The accessibility of medical facilities under various transportation modes such as walking, cycling, and taking public transportation is to be explored in the future. In addition, due to the large amount of data acquisition and high processing difficulty, the dynamic driving travel data crawled in this study are concentrated, and whether they can represent the driving travel pattern in Xi’an is worth further investigation. (3) The medical needs of different types of resident were not considered, and the attributes of residents such as age, gender and income will have an impact on the choice of medical treatment. In the future, a comprehensive index can be calculated based on the indicators that reflect the above factors, and the algorithm can be further improved.

### 4.3. Policy Implications

For historical reasons, most national medical facilities serving the whole country are concentrated in central cities. At the macro level, the local government should consider its own financial strength and the spatial rationality of the location of medical facilities, and plan for a certain number and scale of medical facilities within the region, in view of the uneven distribution of the spatial accessibility of medical facilities in Xi’an. At the same time, the government should strengthen the supervision and management of basic medical facilities. First of all, regular mapping and management of the service situation of the community basic medical facilities service sites to avoid a large number of community medical sites are not fully used. To pay attention to the demand for medical care of the population in the residential area, we should take both supply and demand as the guiding mechanism, and pay attention to the training of medical and nursing staff and the timely increase in relevant facility beds to ensure the balance between the comprehensive supply capacity of facilities and the demand for medical care of the residential population.

## 5. Conclusions

Based on the population data of Xi’an residential district, this study classifies the search radius according to the hospital level, and improves the 2SFCA model by comprehensively considering the service capability of the hospital and introducing the selection probability function to calculate the spatial accessibility of Xi’an medical facilities. Then, on the basis of accessibility, the accessibility results were evaluated from both spatial and data aspects, and the conclusions shows that: (1) There are significant differences in the services provided by Xi’an medical facilities to subdistricts, and the accessibility gradually decreases from the central urban area to the periphery. The accessibility is generally better in the central urban area, and there is an obvious north-south difference in the surrounding areas. (2) In Xi’an, the unfairness of the accessibility space of medical facilities is obvious. About 81.64% of people having access to 54.88% of medical resources. Based on the Lorenz curve and the corresponding Gini coefficient, it is found that there are obvious differences in the level of medical facilities allocation among subdistricts. Beilin District and Yanta District located in the central circle have higher level of medical resources allocation and the residents enjoy better medical services, followed by Lianhu District and Yanta district in the southern periphery, and their internal medical resources distribution is relatively fair. The distribution of medical facilities in the outer ring of Weiyang District and Baqiao District in the northern periphery is not consistent with the population distribution.The results of this study can help to understand the spatial accessibility of medical facilities in Xi’an and provide a reference for scientific and effective planning, reasonable location, and layout of medical facilities.

## Figures and Tables

**Table 1:** Comparison of four common interpolation methods in ArcGIS.

Interpolation Method	Corresponding Parameter	MEAN	RMSE
Trend	Linear	−0.045	5.384
IDW	The power of the distance is 2	0.085	5.132
Splines	Regular spline function	0.064	4.536
Kriging	Ordinary Kriging	−0.037	4.324

**Table 2:** The correspondence between the Gini coefficient and equity.

Gini	0–0.2	0.2–0.3	0.3–0.4	0.4–0.5	0.5–1
Equity	Absolutely fair	Relatively fair	Relatively reasonable	A bit unfair	Very unfair

**Table 3:** Gini coefficient of accessibility of medical facilities in each district.

Administrative District	Gini Coefficient	Administrative District	Gini Coefficient
Xincheng District	0.11	Beilin District	0.16
Weiyang District	0.55	Lianhu District	0.29
Baqiao District	0.62	Yanta District	0.29

**Table 4:** Comparison of different models.

Different Models	Average Value	Minimum Value	Maximum Value	Standard Deviation
A_i_ (Before optimization)	0.009880532	0	0.012077138	0.001442719
A_i_ (Consider the Comprehensive supply quantity)	0.106521123	0	0.125540356	0.012593554
A_i_ (Consider the Comprehensive supply quantity and probability functions)	0.083444099	0	0.104378067	0.012279903

## Data Availability

Not applicable.
